# Effect of cognitive behavioral therapy in mental health and hardiness of infertile women receiving assisted reproductive therapy (ART)

**Published:** 2012-09

**Authors:** Leili Mosalanejad, Anahita Khodabakshi Koolaee, Safie Jamali

**Affiliations:** 1 *Department of Psychiatry, Jahrom University of Medical Sciences, Jahrom, Iran.*; 2 *Department of Psychiatry, University of Social Welfare and Rehabilitation Sciences, Tehran, Iran.*; 3 *Department of Obstetrics and Gynecology, Jahrom University of Medical Sciences, Jahrom, Iran.*

**Keywords:** *Assisted reproductive therapy*, *Cognitive behavioral therapy*, *Mental health*, *Iranian women*, *Hardiness*

## Abstract

**Background:** Infertility is a stressful event that can give rise to psychological difficulties. Now, a wide range of psychosocial interventions for infertile couples has been developed.

**Objective: **Purpose of this study was to determine the effect of group cognitive behavioral therapy (CBT) to reduce stress, anxiety and depression of women undergoing assisted reproductive therapy (ART).

**Materials and Methods:** This study was an experimental study (before and after study with control group) on infertile women who were referring to Gynecological clinics of Jahrom University of Medical Sciences to receive ART. 31 women who had criteria to enter the study were randomly divided into experimental group (n=15) and control group (n=16). The participants in the experimental group received 1 hour and 30 minute weekly session’s group therapy in 15 week as intervention. For gathering data, depression, anxiety and stress scale (DASS) normalized Persian version and Ahvaz Hardiness Test (AHT) were used to assess psychological distress and psychological hardiness in pre-posttest.

**Results:** There were significant differences in mean score of infertile psychological distress, anxiety, depression, and stress in experimental group pretest with posttest. Furthermore, the results indicated that there were significant differences between hardiness in two groups. The experimental group had higher scores in hardiness than control group (p=0.001).

**Conclusion:** It seems to be, that group therapy interventions, specially, cognitive behavioral therapy (CBT) can be useful and applicable to women who receiving ART.

## Introduction

Infertility is failure to conceive after 1 year of regular unprotected intercourse (1). Demographers tend to define infertility as childlessness in a population of women of reproductive age, while the epidemiological definition is based on ‘trying for’ or ‘time to’ a pregnancy, generally in a population of women exposed to the risk of conception (2). 

Gurunath, Pandian, Anderson and Bhattacharya in their research results that “Based on these estimates and on the current world population, 72.4 million women are currently infertile; of these, 40.5 million are currently seeking infertility medical care” (3). In Iran the prevalence of Infertility among during years was indicted the increasing trend. For example Safarinejad research revealed that “the primary infertility increased significantly from 2.6 to 4.3 to 5.5% for the 1985-1989, 1990-1994 and 1995-2000 marriage cohorts. The prevalence of secondary infertility was 3.4% (95% CI: 2.4-5.1)”. In general, the studies indicated that the prevalence of infertility was raised in the Southern counties than in the Northern countries (4). Infertility is a stressful event that can give rise to psychological difficulties. (5) Increases feelings of anxiety, guilt, somatization and depression (6).

The women who suffer infertility experienced high level of depression, low self-esteem and sexual problems. Even though, the women who under treatment of infertility, when they faced the fail of treatment, the all of hope of recovery was despair immediately (7). These psychological distresses can suffer the ouples. The infertility can be negative impact on self-esteem, the image of their femininity or musicality, and social relationship with the others on couples (8). 

Also depressive and anxiety disorders were highly prevalent among women who visited an assisted reproduction unit (9). Hardiness is defined as a constellation of attitudes, beliefs, and behavioral tendencies that consist of three components: commitment, control, and challenge (10). Hardiness is a constellation of personality characteristics that serves as a resistance resource, when encountering stressful situations. Three basic components comprise hardiness: challenge, which is the perception of change as normal and natural, as well as an opportunity for personal growth; commitment, which is a sense of purpose or meaningfulness in one’s life and a strong involvement in directing one’s life course; and control, which is the belief that one is capable of impacting one’s life circumstances (11).

Many evidences suggest that hardiness positively influences perceptions of stressful life events. In addition, hardiness has a beneficial relationship to self-ratings of physical health and physical symptoms, as well as to depression, anxiety and mental health (12, 13). So far, a wide range of psychosocial interventions for infertile couples has been developed. The suggestion program, which is based on behavioral therapy, cognitive restructuring, emotional self-care, and group support, found to be efficacious in the treatment of the emotional aspects of infertility (14). The goal of this study was to compare the effects of cognitive behavioral therapy on the mental health and hardiness among infertile women.

## Materials and methods

**Participants**

The research method of this project was experiment study (before and after with control group) on 800 infertile women who were referring to Gynecological Clinics of Jahrom University of Medical Sciences to receive ART. The research was implemented by randomized controlled clinical trial. Sampling was from 70 women referred to clinic. All of the participants who had 2-7 years unknown infertility history with no somatic and psychiatric diseases, were Jahrom residents, between 20-35 years of age, and interested to participate in regular group meetings were selected for research. Some of participants announced that they had not time or interest to participating in research. Also, some of them were not proper to participating in study.

**Figure 1:**
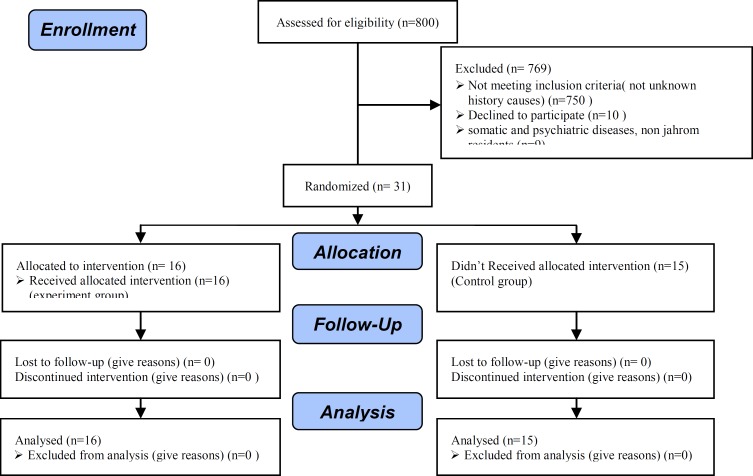
The process of selection of participants

After unification, and selection, only 31 women cooperated to enter the research, which randomly divided into two groups of experimental and control groups. The experiment group (16 women) received 15 sessions of 1 hour and 30 minute weekly cognitive behavioral therapy (CBT) by group therapy in 4 months. The details of sampling process were indicated in figure 1.

The contents of sessions were stress management, negative thought blocking techniques, relaxation therapy and biofeedback, as a behavioral experiments and cognitive approach components entitle cognitive restructuring (It involves recognizing distorted or negative thinking and learning to replace it with more realistic), positive thoughts or beliefs, communication and problem-solving techniques to facilitate expression of emotions and needs and resolution of the infertility crisis, and improving the couple's sexual relationship.

**Exclusion criteria**

- Not meeting inclusion criteria (not unknown history causes).
- Declined to participate.
- Somatic and psychiatric diseases, non Jahrom residents.
- No interest to participating in research.

**Materials**

- Identify goals and rules of meeting, familiarity of members with each other (1 session).
- Understanding capabilities of stress and related factors, relaxation technique (1 session).
- Recognizing distorted or negative thinking, also the effect of these factors to physical, mental and social aspects, relaxation technique (2 sessions).
- How to change the thought and replace them by positive thinking, written task for emotional catharsis, ART therapy, and relaxation technique (2 sessions).
- Negative thought blocking techniques, imagination and biofeedback, relaxation technique (3 sessions).
- Communication and problem-solving techniques to facilitate expression of emotions and needs and resolution of the infertility crisis, relaxation technique (2 sessions).
- Improving the couple's sexual relationship back to philosophy of marriage and cohabitation, relaxation technique (2 sessions).
- The authors’ reached the issues of infertility and new technique management to participants (1 session).
- Provide a brief summary of before session (1 session).

For the control group data gathering was from first visit and three months away from each other with no intervention. To provide research ethics, we designed one session to transfer all of necessary information about new medical treatment to control group after finishing the post-test questionnaires.

**Measurement**

For gathering data the DASS test was used. This test was the short-form Depression Anxiety Stress Scales (DASS-21), which served as the reference standard. The DASS-21 is a 21-item instrument designed to measure the 3 negative affective states of depression, anxiety, and stress. 

In this research, researchers applied the cutoff scores suggested by Lovibond and Lovi-bond. They were reported that “the psychometric properties of the DASS have been extensively evaluated, and there is evidence for the convergent and discriminative validity of data obtained with the instrument” (15). For measuring level of hardiness Ahvaz Hardiness Inventory with 27 questionnaires in 4 point (never to always), were used. Ahvaz hardiness scale is a self-report paper-pencil questionnaire, which is 27 items for measuring personality hardiness. Subjects have one of four options: "never," "rarely," "sometimes", "often”, scoring values are 0, 1, 2, 3, and score range of 0-81. Obtaining a high score on this questionnaire indicate that the person is in psychological hardiness. Cronbach's alpha coefficient has demonstrated for the total hardiness measure for the commitment (0.84), female (0.85), and male (0.84), similar internal consistency coefficients are seen with other samples (16). 

**Statistical analysis**

After collecting data in two groups, descriptive statistics used to evaluate distribution of data and analytical statistics such as student t-test for differences between pretest-posttest in two groups and paired t-test for differences pretest-posttest in each groups using SPSS 18 software. The p-value was determined for research (p<0.5). 

## Results

The age range of samples were 20-25 years (9.67%), 26-31 years (77.42%) and 31-35 years (12.95%). 9.67% of them were employed and the other were housewives (90.33%). 12.91% had a bachelor's degree, 70.96% of them graduate from high school and 16.13% of others had primary to medium school education. 

Results showed that there was significant differences between DASS test in level of stress (p=0.000), anxiety (p=0.001) and depression (0.007) in treatment group pretest with posttest. Therefore the level of psychological distress in experimental group was decrease after intervention (Table I). There was not a significant difference in other group’s pretest-posttest. No significant differences reported between two groups. There were not significant differences between psychological distress after intervention in two groups (p<0.05) (Table II). 

Resent study showed that coping strategies in two groups were changed, but this variation in treatment group was significant after intervention, as hardiness level in treatment group was higher than other group. There was no significant differences between level of hardiness in two groups (p<0.014) (Table III).

**Table I:** The rate of psychological distress in treatment group based on before and after intervention

**State**	**Before**		**After**		**T- student**	**p-value**
	**Mean**	**SD**	**Mean**	**SD**		
Stress	14.64	± 4.07	6.7	± 4.22	5.70	0.001
Depression	13.11	± 4.76	6.41	± 3.26	4.05	0.001
Anxiety	11.11	± 4.45	7.17	± 3.84	3.06	0.007

**Table II:** The rate of DASS score in treatment and control group based on before and after assessment

**DASS**		**Treatment**		**Control**		**T-student**	**p-value**
		**Mean**	**SD**	**Mean**	**SD**		
Pre test							
	Stress	14.6	± 4.07	9.05	± 4.35	0.117	0.88
	Depression	13.11	± 4.76	6.72	± 4.37	0.219	0.24
	Anxiety	11.11	± 4.45	7.72	± 3.96	0.697	0.23
Post test							
	Stress	6.76	± 4.22	7.94	± 3.70	0.21	0.38
	Depression	6.41	± 3.26	5.66	± 3.72	1.39	0.53
	Anxiety	7.17	± 3.84	7.16	± 4.60	1.47	0.99

**Table III:** The rate of hardiness in control and treatment group before and after assessment

**State**	**Treatment**		**Control**		**T-student**	**p-value**
	**Mean**	**SD**	**Mean**	**SD**		
Before	46.82	± 16.04	43.44	± 2.59	2.11	0.42
After	52.41	± 2.36	40.72	± 2.33	0.021	0.001

## Discussion

The present study’s results indicated that there were significant differences between levels of psychological hardiness in treatment group after intervention. In addition, the results suggested that certain coping strategies and hardiness may contribute to illness-resistance in the face of high life stress. Higher scores on hardiness and approach coping predict lower scores on stress and symptoms of illness (10).

Hardiness was found to be negatively related to global stress, which is consistent with previous research linking hardiness to perceptions of global stress and stressful life events (12). These findings, coupled with prior research showing the relation between hardiness to perception of stressful life events and role of psychosocial intervention to alter it. There was also an association between hardiness to other physical and mental health indicators.

Previous research related an approach oriented coping style and hardiness to less global stress (19). Also, the findings were consistent of the other results of researchers who showed the relationship between hardiness and increase level of mental health by decrease psychological distress on infertile women.

Present study indicated that CBT effected on psychological distress. As the findings showed that a mean score of stress, anxiety and depression in experimental group was lower than other group. In addition, results showed that coping strategies in two groups were changed, but this variation in treatment group was increased after intervention, while in the control group had a decreasing trend. Once developed, these individual factors serve a protective function in mitigating the relationship between stress and psychological functioning.

Many evidences emphasis the multiple aspect of CBT on infertile women psychological function as some of them reported that psychological intervention in 14% of cases is conducive to spontaneous pregnancy and that it can be a consequence of reduced stress (20). Present study showed that the hardiness increases as levels of psychological stress decrease.

In one prospective study, women who received psychological interventions had statistically significant higher pregnancy rates, compared with women who received usual care. The result indicates that psychosocial interventions improve pregnancy rates in infertile women (21). For the implemented the research by randomized controlled clinical trial, 89 depressed infertile women that they were under treatment was divided into three groups including; cognitive behavioral therapy, drug therapy (antidepressant medication), and a control group. 

Hämmerli *et al* showed that group and individual/ couple psychotherapy led to a decrease in feelings of anxiety. Upon termination of psychotherapy, a reduction of depressive symptoms in patients was greater after 6 months. Psychotherapy accompanying IVF treatment yielded similar conception success rates to psychological interventions administered to patients not in specific medical care (23).

This study confirms our results about effectively of CBT to decrease psychological distress and usefulness of this program on infertile mental health and coping with stressful life events. Other reports have also demonstrated that psychiatric and counseling interventions (behavioral, cognitive, psychotherapy) actively changes in marital communication and in communication in different social arenas. Various psychological treatments can often contribute to reducing stress but they do rarely increase the possibility of pregnancy (24).

Also this approach reported the positive impact of psychological counseling for stress relief during and after therapy (25). In sum up, the research findings suggested that the counseling centers must be set up inside the medical centers, so that these programs can be helpful to people with psychiatric problems to promote the mental health of them. Furthermore, the psychological interventions could helps to reduce psychological distress and increase rate of successful treatment among infertile women.

The present research faced some limitations such as; the low number of idiopathic infertility, low interest to participate in the meetings because of cultural problems and lack of acceptance due to infertility which is known as social labels.

## Footnote

***Registration ID in IRCT: IRCT201108277407N3***
